# Novel GIS Based Machine Learning Algorithms for Shallow Landslide Susceptibility Mapping

**DOI:** 10.3390/s18113777

**Published:** 2018-11-05

**Authors:** Ataollah Shirzadi, Karim Soliamani, Mahmood Habibnejhad, Ataollah Kavian, Kamran Chapi, Himan Shahabi, Wei Chen, Khabat Khosravi, Binh Thai Pham, Biswajeet Pradhan, Anuar Ahmad, Baharin Bin Ahmad, Dieu Tien Bui

**Affiliations:** 1 Department of Watershed Sciences Engineering, Faculty of Natural Resources, University of Agricultural Science and Natural Resources of Sari, Sari P.O. Box 48181-68984, Iran; a.shirzadi@uok.ac.ir (A.S.); solaimani2001@yahoo.co.uk (K.S.); roshanbah@yahoo.com (M.H.); ataollah.kavian@yahoo.com (A.K.); khabat.khosravi@gmail.com (K.K.); 2 Department of Rangeland and Watershed Management, Faculty of Natural Resources, University of Kurdistan, Sanandaj 66177-15175, Iran; k.chapi@uok.ac.ir; 3 Department of Geomorphology, Faculty of Natural Resources, University of Kurdistan, Sanandaj 66177-15175, Iran; h.shahabi@uok.ac.ir; 4 College of Geology & Environment, Xi’an University of Science and Technology, Xi’an 710054, China; chenwei0930@yahoo.com; 5 Institute of Research and Development, Duy Tan University, Da Nang 550000, Vietnam; phambinhgtvt@gmail.com; 6 Centre for Advanced Modelling and Geospatial Information Systems (CAMGIS), Faculty of Engineering and IT, University of Technology Sydney, Sydney, NSW 2007, Australia; Biswajeet.Pradhan@uts.edu.au; 7 Department of Energy and Mineral Resources Engineering, Choongmu-gwan, Sejong University, 209 Neungdong-ro, Gwangjin-gu, Seoul 05006, Korea; 8 Department of Geoinformation, Faculty of Geoinformation and Real Estate, Universiti Teknologi Malaysia (UTM), Skudai 81310, Malaysia; anuarahmad@utm.my (A.A.); baharinahmad@utm.my (B.B.A.); 9 Geographic Information Science Research Group, Ton Duc Thang University, Ho Chi Minh City, Vietnam; 10 Faculty of Environment and Labour Safety, Ton Duc Thang University, Ho Chi Minh City, Vietnam

**Keywords:** landslide, alternating decision tree, GIS, machine learning algorithms, Iran

## Abstract

The main objective of this research was to introduce a novel machine learning algorithm of alternating decision tree (ADTree) based on the multiboost (MB), bagging (BA), rotation forest (RF) and random subspace (RS) ensemble algorithms under two scenarios of different sample sizes and raster resolutions for spatial prediction of shallow landslides around Bijar City, Kurdistan Province, Iran. The evaluation of modeling process was checked by some statistical measures and area under the receiver operating characteristic curve (AUROC). Results show that, for combination of sample sizes of 60%/40% and 70%/30% with a raster resolution of 10 m, the RS model, while, for 80%/20% and 90%/10% with a raster resolution of 20 m, the MB model obtained a high goodness-of-fit and prediction accuracy. The RS-ADTree and MB-ADTree ensemble models outperformed the ADTree model in two scenarios. Overall, MB-ADTree in sample size of 80%/20% with a resolution of 20 m (area under the curve (AUC) = 0.942) and sample size of 60%/40% with a resolution of 10 m (AUC = 0.845) had the highest and lowest prediction accuracy, respectively. The findings confirm that the newly proposed models are very promising alternative tools to assist planners and decision makers in the task of managing landslide prone areas.

## 1. Introduction

Landslides, which are very frequent natural hazards in mountainous regions, cause serious damages to economy and human lives. In past decades, tremendous efforts have been made to predict landslides for efficient hazard management. However, it is difficult to perfectly predict the natural mechanism of landslides, as they are controlled by many conditioning factors. Many methods have been developed and applied for spatially predicting landslides in recent years, which can be grouped into two main types, namely qualitative methods and quantitative methods [1]. Qualitative techniques (landslide inventory and weighted methods) are based on the judgment of experts, whereas quantitative techniques (statistical, probabilistic and deterministic methods) are based on mathematical objective algorithms [2]. In general, quantitative methods often produce better results compared with qualitative methods [3,4]. For example, Borrelli et al. [5] used a bivariate statistical model for landslide modeling and achieved reasonable results and Ciurleo et al. [6] and Cascini et al. [7] compared heuristic, statistical and deterministic methods. Their results depicted that deterministic methods are slightly better than the other models. Other scientists have recently proposed several methods based on physical modeling, suggesting that they may be more accurate because they use expressions based on universal physical laws. Furthermore, analysis of past landslides may give useful data which can be used in methods based on physical modeling [8,9,10,11,12,13].

In recent years, machine learning algorithms have been popularly used for developing quantitative models for spatially predicting landslides. Ada and San [14] applied and compared different machine learning methods, namely support vector machines (SVM) and random forest (RF), for landslide susceptibility mapping, and stated that these machine learning methods are promising for landslide prediction and modeling. Other machine learning methods, namely Fisher’s Linear Discriminant Analysis (FLDA), Bayesian Network (BN), Logistic Regression (LR), and Naïve Bayes (NB), were applied and compared by Pham et al. [15]. Goetz et al. [16] compared several machine learning algorithms including Random Forest (RF), SVM, and LR with some conventional statistical prediction techniques, namely weights-of-evidence (WOE) and generalized additive models (GAM), and revealed that machine learning methods, especially RF, are more powerful in spatial prediction of landslides. Even though many machine learning methods have been developed and applied in recent decades, the development and application of new techniques and algorithms is still needed for enhancing the quality and accuracy of landslide prediction. More recently, the performance of single machine learning methods is frequently improved using several ensemble techniques such as AdaBoost, MultiBoost, Bagging, and Rotation Forest [17]. Ensemble techniques utilize multiple learning algorithms to combine different machine learning methods for generating hybrid models; thus, they can efficiently handle complex input to produce a better output [18,19,20,21].

The main purpose of this study was to evaluate the efficiency of several ensemble techniques (MB, BA, RF, and RS) in improving the performance of a base classifier, namely Alternating Decision Trees (ADTree). The difference between this study and earlier studies is using two scenarios of the combination of sample size and raster resolution, including 60%/40% and 70%/30% with a raster resolution of 10 m, and 80%/20% and 90%/10% with a raster resolution of 20 m for preparing landslide susceptibility mapping (LSM) and assessing their performance by ADTree algorithm and its four ensembles. For this, some shallow landslides around Bijar City, Kurdistan Province, Iran, were collected and considered as database for modeling. Various criteria including statistical indexes, the receiver operating characteristic (ROC) curve and the Friedman and Wilcoxon tests were applied for validation of the developed models.

## 2. Description of Study Area

**Figure 1:**
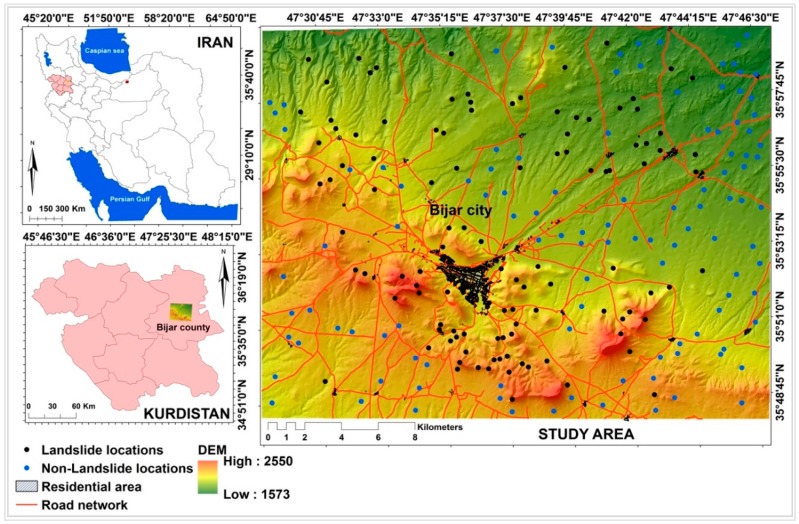
Location of landslides in the study area in Kurdistan Province of Iran.

In this study, a region around Bjar City in the eastern part of the Kurdistan Province in Iran, which is hit by many shallow landslides, was selected. Geographically, it is located between latitudes of 35°48′25″ N and 35°59′50″ N, and longitudes of 47°28′50″ E and 47°46′44″ E, covering an area of about 598 km^2^ (Figure 1). In terms of topography, the elevation of the study area mostly covers low land and plain ranging between 1573 m and 2550 m above sea level, with an average of 1898 m. Slope angles vary from 0° to 60° such that most of the area is mainly hilly. According to the Köppen climatic classification, the study area has a cold climate (type D). In addition, the minimum and maximum annual average temperatures are 4.4 °C and 13.4 °C, respectively. The analysis of rainfall data from various gauge stations between 1987 and 2010 shows that the annual average precipitation is about 338 mm. Additionally, analysis of Intensity–Duration–Frequency curve (IDF) of the synoptic station of Bijar City reveals that the precipitation with a low duration, for example 15 min, creates a high precipitation intensity of 33.661 mm/h, for a 20-year return period resulting in frequent shallow landslides in the study area. The number of frost days is 104, and the number of snow days is 35 [22].

**Figure 2:**
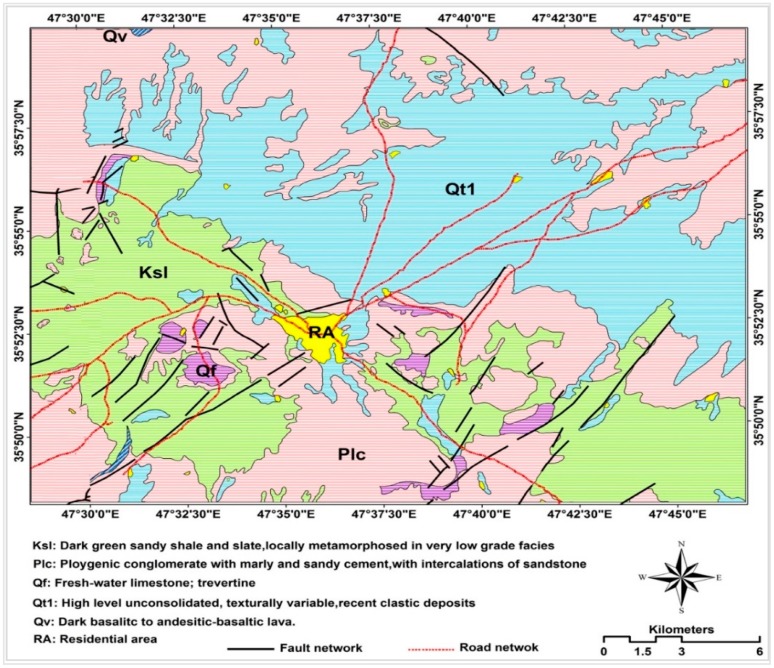
Lithological map of the study area.

In terms of land cover, most of the area, 78%, has been covered by dry-farming lands while barren lands cover the lowest amount. However, other classes of land covers including irrigated lands, wood lands, pasture lands, residential areas, and barren lands are existant and classified. In terms of geology, most of the area, 94%, consists of conglomerate and siltstone with intermediate shale and marl while 6% is covered by volcanic rocks. The study area belongs to the Sanandaj-Sirjan tectonic zone where most of mountains have been formed by carbonated stones (Miocene formation) while the hilly areas have mainly been covered by Pliocene formations including shale and marl, and quaternary deposits [22] (Figure 2). Overall, precipitation with a high intensity and a low duration in conjunction with loose and discontinuity formation of hilly mountains can be considered as the most reason for occurring landsides in the study area.

## 3. Data Acquisition and Processing

### 3.1. Landslide Inventory Map

Shirzadi et al. [22] reported that a landslide inventory map (LIM) is prerequisite information for landslide susceptibility modeling. Additionally, Galli et al. [23] indicated some objectives of LIM for modeling landslides including: (1) detection and monitoring of location and type of landslides; (2) the frequency of landslides occurrence; (3) detection and monitoring of single triggering events such as earthquakes, intense rainfall and rapid snowmelt; (4) the frequency–area analysis of slope instability; and (5) required information for landslide susceptibility or hazard maps [23]. Thus, a reliable and accurate LIM, which is more concordant with region reality, can obtain a reasonable result in landslide modeling. The locations of 111 shallow landslides were firstly obtained from the Forests, Rangeland and Watershed Management Organization of Iran (FRWOI), and were then checked using field surveys, interpretations of aerial photographs (1:40,000 scale) and Google Earth images [22,24]. In this study, two scenarios were used: (1) the combination of sample sizes of 60%/40% and 70%/30% with a resolution of 10 m; and (2) the combination of sample sizes of 80%/20% and 90%/10% with a resolution of 20 m. These scenarios were selected after changing different sample sizes and raster resolutions in this study.

Accordingly, from the 111 landslide locations, the number of landslides for sample size of 60%/40% is 67 and 44 landslides for training and validation dataset. These values for sample size of 70%/30% and 77 and 34, for sample size of 80%/20% are 89 and 22 and for sample size of 90%/10% are 99 and 12, respectively. A comprehensive study of landslide inventory of the study area can be seen in [22]. They cited that most shallow landslides have a depth lower than 3 m in which rotational sliding, complex and rotational falling assigned 70.6%, 22.4% and 6.3% of the landslides, respectively. In addition, they reported that, in addition to precipitation and geological configuration, man-made factors including land use change and cutting the foot of slopes were the most common causes of landslide incidence. Additionally, results of field surveys reveal that landslide length ranged between 70 and 280 m. Moreover, landslide widths ranged between 7 and 293 m. The average, median, mode, standard deviation, and skewness of the landslide length were 36.388, 25.800, 14.50, 34.003, and 3.738 m, respectively. Statistical indices such as average, median, mode, standard deviation, and skewness for landslide widths were 62.721, 43.300, 31, 54.821, and 2.703 m, respectively [22].

### 3.2. Landslide Conditioning Factors

As landslide occurrence is a function of geo-environmental factors which is different from one region to another, the contributions of these factors in landslide incidence are also completely different [19]. The conditioning factors of the present study were selected considering many existing landslide susceptibility studies and data availability in the study area. In this regard, twenty landslide conditioning factors were adopted in five categories: (1) topographic factors (slope, aspect, elevation, curvature, plan curvature, profile curvature, and sediment transport index (STI)); (2) hydrological factors (rainfall, annual solar radiation, stream power index (SPI), topographic wetness index (TWI), distance to rivers, and river density); (3) lithological factors (lithology, distance to faults, and faults density); (4) land cover factors (land use and normalized difference vegetation index (NDVI)); and (5) anthropogenic factors (distance to roads and road density) (Table 1). In this study, two raster resolutions of 10 m and 20 m for all conditioning factors in conjunction with four sample sizes, namely 60%/40%, 70%/30%, 80%/20% and 90%/10%, were used in landslide modeling process.

Slope is a vital conditioning factor that is widely used for landslide susceptibility modeling [25]. The slope values were extracted from the digital elevation model (DEM) prepared from advanced space borne thermal emission and reflection radiometer (ASTER) satellite image with resolution of 30 m × 30 m [26,27] and classified into eight classes (Table 1). The correlation between slope aspect and occurrence of landslides is evident [28]. Different aspects affect the soil and rock degrees of weathering and the moisture content. The aspect map was extracted from the DEM and reclassified into nine directions (Table 2). The variety of elevation has a significant influence on landslide [29,30]. Curvature reflects the shape of ground surface which in turn affects the occurrence of landslide [22]. The map of curvature for the study area was generated from DEM in six classes (Table 1). The plan curvature values reflect the steepness degree of slopes influencing the characteristics of surface runoff [29]. The plan curvature values were derived from the DEM and classified according to the natural break method, into six classes (Table 1). Calvello and Ciurleo [31] demonstrated that natural breaks is the best classification criteria (of the two adopted by the authors) to be used in classifying the variables. Profile curvature is the curvature in the vertical plane parallel to the slope direction [32]. The values of profile curvature acquired through DEM and ArcGIS Tools were divided into six classes using natural break method (Table 1). The sediment transport index (STI) indicates the amount of sediment transported by overland flow. This hydrological factor is based on the catchment evolution erosion theories and the transport capacity limiting sediment flux [33]. The STI is calculated from the following formula:

(1) $$STI={\left(\frac{A_{s}}{22.13}\right)}^{0.6}{\left(\frac{\sin\beta }{0.0896}\right)}^{1.3}$$

where $A_{s}$ is specific catchment area (m^2^) and sinβ is slope gradient (radian) [34].

In this study, STI map was divided into six classes (Table 1). Generally, rainfall plays a critical role in the occurrence of shallow landslides. Based on the rainfall data of the study area, the values of rainfall were divided into seven categories (Table 1). Annual solar radiation is defined as the mean solar radiation converged at a given pixel within one year [34]. The lower the annual solar radiation is, the higher the probability of failure occurrence will be, due to less available pore spaces of soil [34]. It is computed based on the aspect and slope by ArcGIS 10.2 using “Area Solar Radiation” command. The solar radiation map in this study was reclassified into seven classes with natural break intervals (Table 1). The stream power index (SPI), a factor being able to measure the intensity and erosive power of slope surface runoff, was calculated as [35]:

(2) $$SPI=A_{s}\tan\beta$$

where *A_s_* is the specific catchment area (m^2^), and *β* represents the local slope gradient (radian). The values of SPI are determined by the characteristics of underlying soil and runoff. Eventually, the SPI map manifested six intervals, as shown in Table 1. Topographic wetness index (TWI) is used to quantitatively evaluate the tendency of runoff and the position where water converges [35]. The TWI values were calculated as:

(3) $$TWI=\ln(\frac{\alpha }{\tan\beta })$$

where *α* is the cumulative upslope area draining through a point (m^2^) and *β* is the slope angle (radian) at the point. In this study, the TWI map was composed of six categories (Table 1). Distance to rivers is one of the conditioning factors that have an effective role in landslide stability [36]. Hence, it should be considered for landslide modeling [37]. It was generated in five classes (Table 1). Another important conditioning factor used for landslide susceptibility mapping by many researchers is river density [30]. The river network map in this study was reclassified into seven classes with natural break intervals method (Table 1). Lithology is a basic factor determining the geotechnical engineering characteristics. The soil and rock that have low engineering characteristics always have a potential for surface sliding. According to the lithological map extracted from Sanandaj geological map with the scale of 1:100,000, the lithology was reclassified into three categories (Table 1).

Moreover, the distance to faults map with six categories (Table 1) was constructed from the fault lines of the lithological data with the help of ArcGIS 10.2. In addition, the degree of influence of faults was measured by fault density arranged into seven classes, as shown in Table 1. Different land types have different permeability and strengths which are closely related to the stability of slopes. Land use was classified into five classes by means of aerial photos interpretation and supervised classification method (Support Vector Machine algorithm) (Table 1). NDVI can qualitatively assess the vegetation coverage condition of slope surfaces. The NDVI is calculated from reflectance measurements in the red and near infrared (NIR) portion of the spectrum as [37]:

(4) $$\mathrm{NDVI}=\frac{\left(\mathrm{NIR}(\mathrm{Band}4)-\mathrm{Red}(\mathrm{Band}3)\right)}{\left(\mathrm{NIR}(\mathrm{Band}4)+\mathrm{Red}(\mathrm{Band}3)\right)}$$

where Red and NIR stand for the spectral reflectance measurements acquired in the red and near-infrared regions, respectively. The NDVI varies between 1 and −1, and its map was classified into seven classes (Table 1). Distance to road is another critical factor that is widely used in landslide risk assessment models. Literature shows that a large number of landslides was observed closer to the roads [22]. It was mapped with five categories in this study (Table 1). Furthermore, for estimating the effects of road engineering, the road density was employed as a landslide conditioning factor. The road density layer in the study area was generated with seven categories (Table 1).

## 4. Methodology

### 4.1. Alternating Decision Tree (ADTree)

ADTree is one of the most successful classification algorithms, which is widely applied in data mining. ADTree algorithm was proposed by Freund and Mason [38] in 1993 based on Boosting. ADTree base-classifier can not only generate classification results, but also provide the confidence of results, which is employed to evaluate the accuracy of results [39]. This algorithm is composed of prediction nodes and decision nodes [40]. The elements in a training set can be divided into prediction nodes by split tests and the corresponding predictive values of prediction nodes are obtained. Furthermore, with iterative computation, growing and pruning, the ADTree base-classifier is generated, which has a favorable applicability to deal with complex and enormous database [41]. Assuming is a split testing of predictive node, we get Equation (5):

(5) $$Z(c)=2(\sqrt{W_{+}(c)W_{-}(c)}+\sqrt{W_{+}(-c)W_{-}(-c)})+W^{\prime }$$

where $W_{+}(c)$ and $W_{-}(c)$ are the weighted sum of positive tuples and negative tuples that meet the demand of c. $W^{\prime }$ is other tuples’ weighted sum except the tuple sets divided into p. By finding the minimum value of Z, the best split testing can be obtained [42]. The optimal construction algorithm of ADTree, which utilizes the $Z_{\mathrm{pure}}$ pruning technology, was invented by Pfahringer [43] (Equation (6)).

(6) $$Z_{\mathrm{pure}}=2(\sqrt{W_{+}}+\sqrt{W_{-}})+W^{\prime }$$

where $Z_{\mathrm{pure}}$ is the low limit of Z, which can be used for cutting the evaluation of some predictive nodes.

### 4.2. Bagging (BA)

Bagging is an ensemble of various component learners [44]. Essentially, various data subsets are acquired by repeated sampling, and the extensiveness and otherness of component learners rise significantly through training the data subsets mentioned above [45]. In addition, the independence of component learners is relatively excellent, and different algorithms can be run as parallel. According to the core idea of Bagging, the main process of this algorithm includes: (1) selecting data randomly and independently from original data; (2) repeating Step (1) several times to generate a certain amount of independent datasets; (3) designating a weak learning algorithm to learn various datasets; (4) obtaining the sequence of predictive function; and (5) voting for the results and selecting the result with the most votes as a final result [46]. As a sort of ensemble learning method, Bagging can weaken the defects of component learners and raise the recognition rate of unstable classifiers. Therefore, Bagging has been widely combined with various weak classifiers, such as Random Forest that combines Bagging and Decision Tree [47]. Sequences of algorithms related to Bagging have been employed to build landslide susceptible models [48,49,50].

### 4.3. Multiboost (MB)

Multiboost belongs to classification ensemble algorithms, which are made up of various classifiers generated through classification learning. Due to the diversity among classifiers, the classification errors decrease dramatically. The errors of base classifiers can be calculated by Equation (7).

(7) $$e=\frac{\sum_{x_{j}\in S^{\prime },C_{t}(x_{j})\neq y_{j}}\mathrm{weight}(x_{j})}{m}$$

where e is the errors of base classifiers; $S^{\prime }$ is the dataset; and $x_{j}$ and $y_{j}$ are the elements of datasets. Practically, Multiboost is an organic combination of Wagging and Adaboost which are representative classification ensemble techniques [51]. The main idea of Multiboost is that Wagging and Adaboost can reduce the variance and deviation. Hence, the precision of classification results can be improved further. In the Multiboost algorithm, various classifier-based models are first constructed using training subsets. The weights of classifier-based models are then adjusted to optimize classification accuracy [49]. In view of the advantages of Multiboost mentioned above, this algorithm has been applied in wider research fields [52,53].

### 4.4. Random Subspace (RS)

RS was proposed by Ho in 1998 [54]. As another important ensemble learning method, RS also has a superior generalization performance compared with traditional component learners. The definition of subspace can be expressed as follow: Assuming W is the nonempty subset of linear space V, when Equations (8) and (9) hold, W is the linear subspace of V.

(8) α+β∈W(α,β∈W)

(9) kα∈W(α∈W,k∈R)

where R represents the real number field and k is a number of R. The base learners of RS are formed by randomly sampling feature subsets, and RS is more suitable to analyze high-dimensional data [55]. The base learners only learn parts of sample information from various feature subsets. Therefore, to utilize complete sample information, multiple learners should be fused organically. Specifically, the feature subspaces are picked out using Bootstrap Method. On this basis, multiple base learners can be generated by classification algorithms using machine learning methods. Finally, various base classifiers can be bound together in accordance with majority voting method or multiplication rules. In recent years, numerous research achievements have embodied the excellent classification performance of RS [56,57,58].

### 4.5. Rotation Forest (RF)

**Figure 3:**
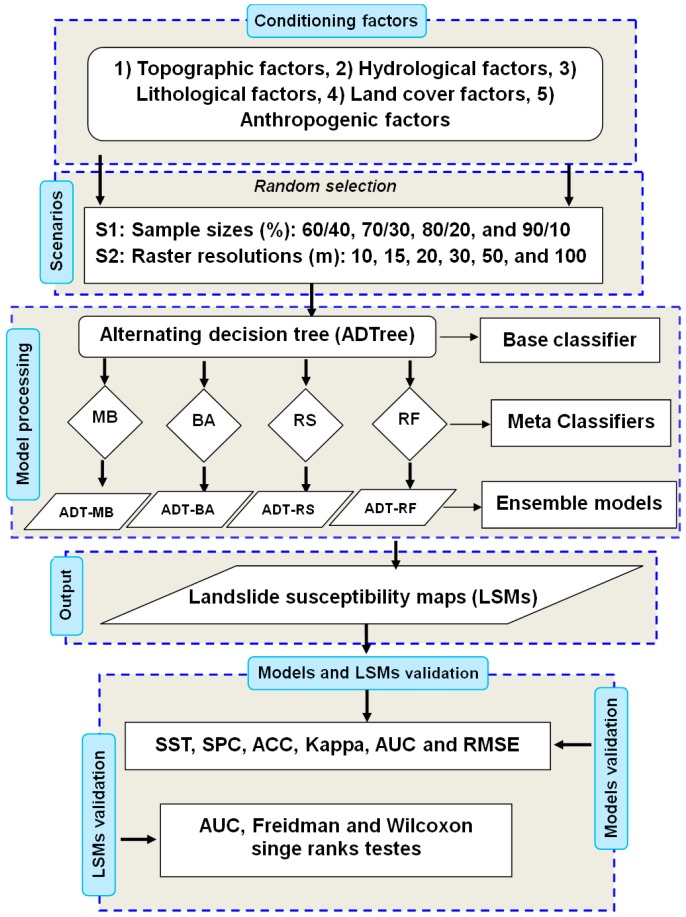
Flowchart of modeling process and methodology used in this study.

The RF algorithm is used to promote the difference and accuracy of base classifiers based on feature transformation [59]. Before selecting subsamples, the sets of sample attributes should be segmented and combined randomly to obtain sequences of subsets of sample attributes, of which data can be preprocessed by feature transformation. Compared with Random Forest algorithm, which is the basis of RF, RF algorithm has a better performance on processing high dimensional and small-sample database [60,61,62]. The main procedure of building RF model includes: (1) dividing the attribute sets into several subsets; (2) obtaining sample subsets by resampling and making feature transformation on subsets of sample attributes; (3) realigning the rotation matrix according to sequence of original attribute sets; (4) training base classifiers based on the data which have been rotated; and (5) integrating results of various base classifiers and outputting the final forecast category. The probability of a sample belonging to one category can be calculated by Equations (10) and (11).

(10) $$u_{\omega }(x)=\frac{\sum_{i=1}^{L}d_{i,j}(xR_{i}^{a})}{L}(j=1,\cdots ,c)$$

(11) $$x=\arg\max(u_{\omega }(x))(\omega \in C)$$

where *x* is a classification sample; ω is one of the categories; *C* is the universal set of categories; *L* is the total number of base classifiers; $R_{i}^{a}$ represents the rotation matrix. The flowchart of methodology is shown in Figure 3.

### 4.6. Comparison and Validation Techniques

#### 4.6.1. Statistical Index-Based Measures

In this study, Sensitivity (SST), Specificity (SPF) and Accuracy (ACC) are popular statistical indexes used for validation of model performance. Out of these, the SST and SPF are the proportion of the landslide and non-landslide instances which are correctly predicted as landslide and non-landslide, respectively [37,63]. Values of these indexes are calculated using the values extracted from confusion matrix as below:

(12) $$\mathrm{SST}=\frac{\mathrm{TP}}{\mathrm{TP}+\mathrm{FN}}$$

(13) $$\mathrm{SPF}=\frac{\mathrm{TN}}{\mathrm{TN}+\mathrm{FP}}$$

(14) $$\mathrm{ACC}=\frac{\mathrm{TP}+\mathrm{TN}}{\mathrm{TP}+\mathrm{NT}+\mathrm{FP}+\mathrm{FN}}$$

(15) $$\mathrm{Kappa}\;\mathrm{index}\;(K)=\frac{P_{C}-P_{\exp}}{1-P_{\exp}}$$

(16) $$P_{C}=\left(\mathrm{TP}+\mathrm{TN}\right)/\left(\mathrm{TP}+\mathrm{TN}+\mathrm{FN}+\mathrm{FP}\right)$$

(17) $$\mathrm{Pexp}=\left(\left(\mathrm{TP}+\mathrm{FN}\right)\left(\mathrm{TP}+\mathrm{FP}\right)+\left(\mathrm{FP}+\mathrm{TN}\right)\left(\mathrm{FN}+\mathrm{TN}\right)/\sqrt{\left(\mathrm{TP}+\mathrm{TN}+\mathrm{FN}+\mathrm{FP}\right)}\right)$$

(18) $$\mathrm{RMSE}=\sqrt{\frac{1}{n}}\sum_{i=1}^{n}(X_{\mathrm{pred}.}-X_{\mathrm{act}.}{)}^{2}$$

where TP (true positive) and TN (true negative) are the number of instances predicted correctly, whereas FP (false positive) and FN (false negative) refer the numbers of instances predicted erroneously. P_c_ is the proportion of number of pixels that have been classified correctly as landslide or non-landslide pixels. P_exp_ means the expected agreements. $X_{\mathrm{pred}.}$ is the predicted values in the training dataset or the validation dataset. $X_{\mathrm{act}.}$ is the actual (output) values from the landslide susceptibility models [20].

#### 4.6.2. Receiver Operating Characteristic Curve

The ROC curve is a popular method usually applied to validate the performance of models in landslide susceptibility assessment. It is constructed by using pairs of two values which are true positive rate and false negative rate [64,65]. Each point on this curve might be related to a specific decision criterion for the prediction accuracy; thus, the ROC curve is very useful for validating the predictive accuracy of models [66,67,68,69]. To quantitatively validate the models, area under this curve (AUC) is often used. More specifically, an ideal model has the AUC value of 1, and better models have higher AUC values [22,70].

#### 4.6.3. Parametric and Non-Parametric Statistical Tests 

Freidman test, which was introduced by Friedman [71], is a common method for validating the performance of models. It is based on the null prior hypothesis that there is no significant difference among the applied models, and then statistical indexes including *p*-values and Chi square values of all models are calculated and ranked. If *p*-values and Chi square values are higher than standard values of 0.05 and 3.841, respectively, then the null prior hypothesis is not true and rejected, and thus, we can conclude that all models are significantly different. The Wilcoxon signed-rank test is often used to validate and compare the models on the base of evaluating the statistical significance of differences among the models. For that, the null hypothesis which is based on the pre-assumption that there is no statistical difference at the significant level of 0.05 between the models, and then the statistical values (*Z* and *p* values) are determined and evaluated. More specifically, as *p* value < 0.05 and *Z* values beyond the critical values (±1.96), then the null hypothesis is not true and rejected, and thus the difference among the models is significant [63].

### 4.7. Factor Selecting based on the Information Gain Ration (IGR) Technique 

Information Gain Ratio (IGR) is a widely used feature selection for landslide conditioning factors in the modeling of landslide susceptibility [49]. It also helps in determining the importance of each factor for modeling so that it suggests the suitable weights assigned for each factor in generating the input datasets. Information Gain (IG) value of factor $x_{i}$ in respective with the output class *y* is determined by calculating the reduction of the entropy in bits as below [49]:

(19) $$\mathrm{IG}(y,x_{i})=E\left(y\right)-E\left(y|x_{i}\right)$$

where $E\left(y|x_{i}\right)$ is inferred the entropy value of y after incorporating the values of factor $x_{i}$ and E(y) is inferred the entropy of y. $E\left(y|x_{i}\right)$ and E(y) are calculated by the following equations:

(20) $$E\left(y\right)=-\sum_{i}Q\left(y_{i}\right)\log_{2}\left(Q\left(y_{i}\right)\right)$$

(21) $$E\left(y|x_{i}\right)=-\sum_{i}Q\left(y_{i}\right)\sum_{j}Q\left(y_{i}|x_{i}\right)\log_{2}\left(Q\left(y|x_{i}\right)\right)$$

where $Q\left(y_{i}\right)$ is defined as the prior probability of y and $Q\left(y_{i}|x_{i}\right)$ is defined as the posterior probabilities of y corresponding to the factor $x_{i}$.

## 5. Result and Analysis

### 5.1. Important Factors for Landslide Modeling

The results of different combinations of training and validation datasets showed that for a combination of 60%/40% with the resolution of 10 m, TWI, slope angle, aspect, LS and profile curvature have demonstrated effective impacts on landslide occurrence. Other factors did not show any effect on the occurrence of landslide in the current study so that they were removed from the modeling process. For three other combinations including 70%/30%, 80%/20% and 90%/10%, the results were different since all the selected conditioning factors had an impact on the landside occurrences.

In the 70%/30% combination, TWI (Average Merit (AM) = 0.597) had the highest impact and profile curvature (AM = 0.042) had the lowest, however in the combinations of 80%/20% and 90%/10%, slope angle (AM = 0.509) had the highest impact on the occurrence of the past landslides. Land use (AM = 0.058) and profile curvature (AM = 0.031) showed the lowest impact on the landslide for the combinations of 80%/20% and 90%/10%, respectively (Table 2). For resolution of 20 m, similar to resolution of 10 m, in the combination of 60%/40%, TWI (AM = 0.142) had the significant impact on the landslide occurrence, followed by slope angle, aspect and LS. Other factors, due to obtaining the AM equal to 0, did not illustrate any impact on the landslide occurrence in the study area. However, in the three other combinations, the results were almost similar to the resolution of 10 m so that slope angle and profile curvature had the highest and the lowest impact on landslide modeling process (Table 2).

The performance of ADTree algorithm using training dataset for the resolution of 10 m showed that the combination of 70%/30% using all statistical measures including SST (0.951), SPF (1.00), ACC (0.975), Kappa (0.950) and RMSE (0.157) had the highest performance compared to the other combinations while the combination of 60%/40% had the lowest effectiveness. Moreover, in 20 m resolution, similar to 10 m resolution, while the combination of 70%/30% had the highest performance shown by SST (0.960), ACC (0.926), Kappa (0.851) and RMSE (0.239), the combination of 80%/20% only had the highest performance in terms of SPF (0.911). Overall, results based on the resolutions of 10 m and 20 m indicated that the combination of 70%/30% (highest goodness-of-fit) demonstrated more performance than the combinations of 80%/20%, 90%/10% and 60%/40% (lowest goodness-of-fit) (Table 3).

The performance of ADTree algorithm using validation dataset is shown in Table 4. Results indicated that, for 10 m and 20 m resolutions, the combination of 90%/10% had more prediction power calculated by SST, SPF, ACC, and Kappa, while the combination of 70%/30% displayed the highest prediction capability for both 10 and 20 m resolutions in terms of RMSE. Additionally, the results indicate that the combination of 60%/40% for both 10 and 20 m resolutions showed the lowest prediction power.

### 5.2. Selecting the Best Raster Resolution for Each Combination

**Figure 4:**
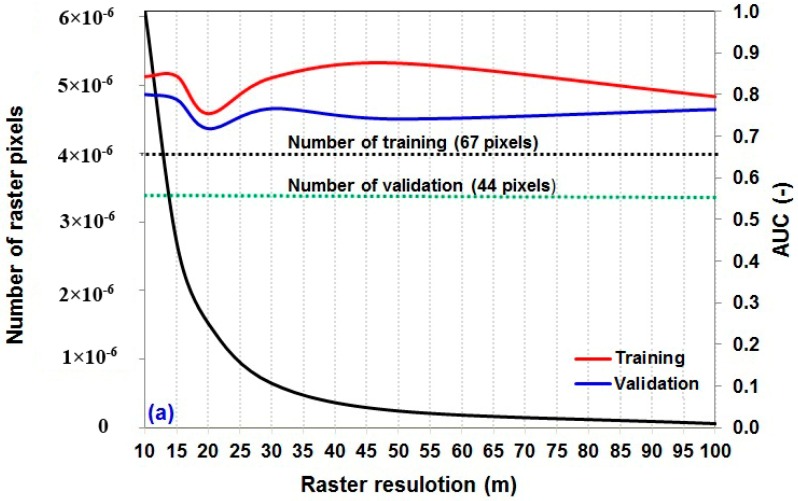

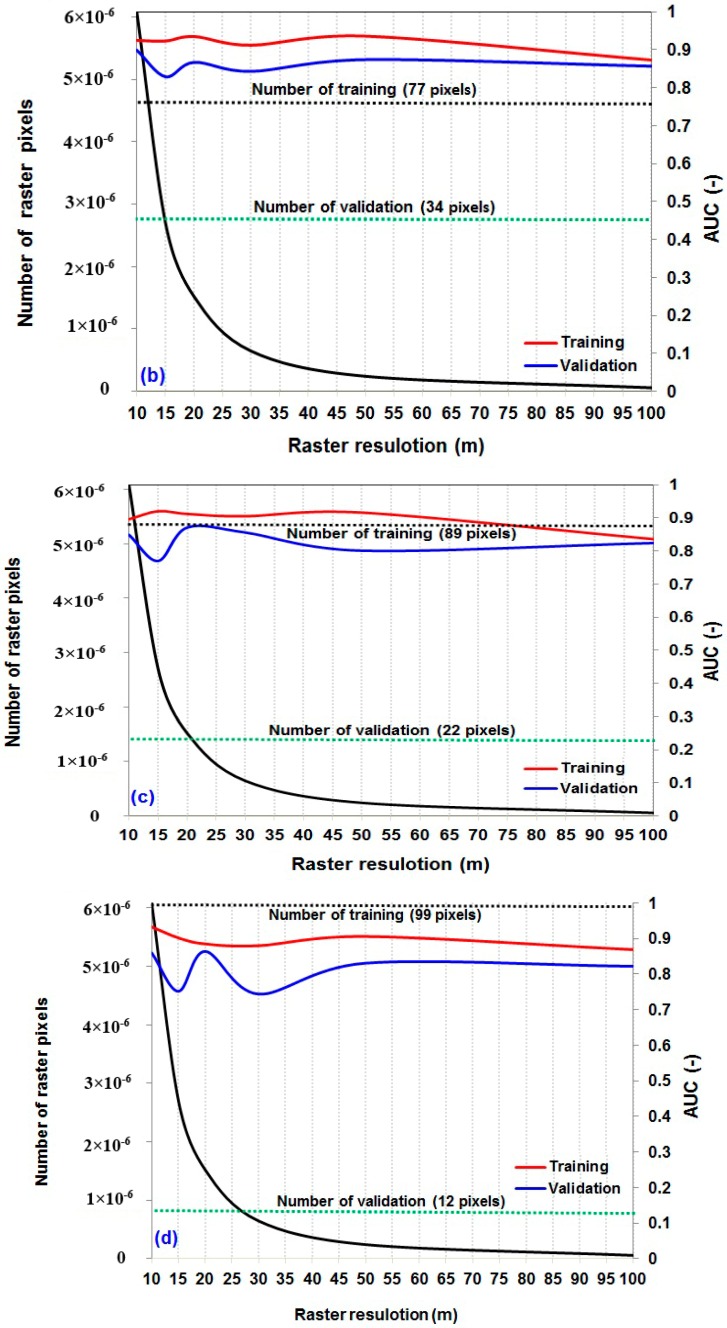
The effects of sample size and raster resolution on the performance of landslide modeling: (**a**) sample size of 60%/40%; (**b**) sample size of 70%/30%; (**c**) sample size of 80%/20%; and (**d**) sample size of 90%/10%.

The prediction of different raster resolutions and sample sizes has been made simultaneously and results have been checked using the AUC for training and validation datasets (Figure 4a–d). Results show that the validation dataset is more sensitive than training dataset in change of pixel resolutions and sample sizes. Basically, in the combinations of 60%/40% (Figure 4a) and 70%/30% (Figure 4b), the resolution of 10 m had the highest goodness-of-fit and power prediction. In addition, these figures reveal that, in the combinations of 80%/20% (Figure 4c) and 90%/10% (Figure 4d), the resolution of 20 m displayed the highest performance using training and validation datasets. The results generally indicated that the combination of 70%/30% with raster resolution of 10 m had the highest and the combination of 60%/40% with raster resolution of 20 m the lowest performance.

### 5.3. Landslide Modeling Process

The best combination of sample size and raster resolution in the modeling process was selected for performing the ensemble models of the ADTree algorithm, namely the MB, BA, RF and RF in both training and validation phases. Basically, for sample sizes of 60%/40% and 70%/30%, the resolution of 10 m was selected while the resolution of 20 m was considered for sample sizes of 80%/20% and 90%/10%. The number of seed and iteration in the landslide modeling process can affect the results of goodness-of-fit and prediction accuracy of the models. The results of selecting the best optimal parameters of ensemble models are shown in Table 5. The effects of number of seed and iteration for both of training and validation datasets and four different sample sizes of 60%/40%, 70%/30%, 80%/20% and 90%/10% have been investigated for all ensemble models (Table 5).

**Figure 5:**
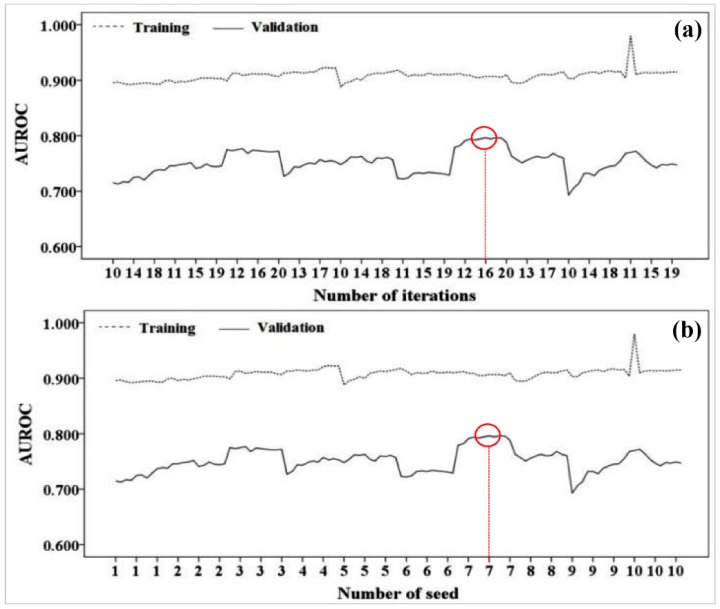

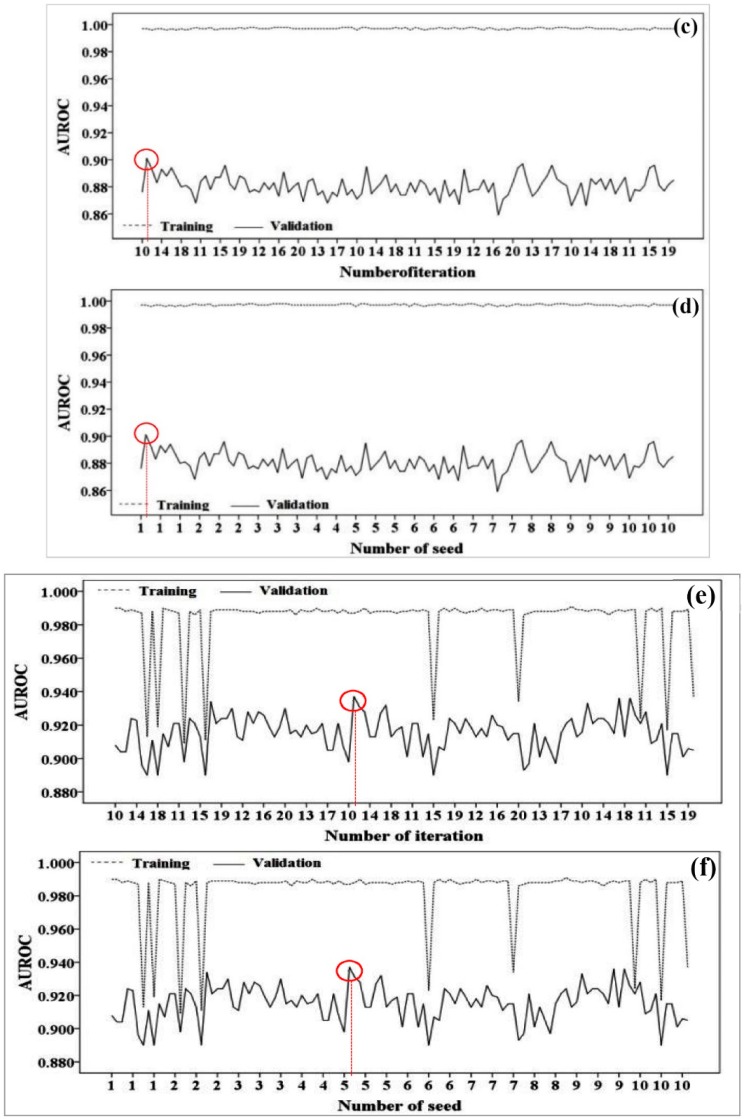

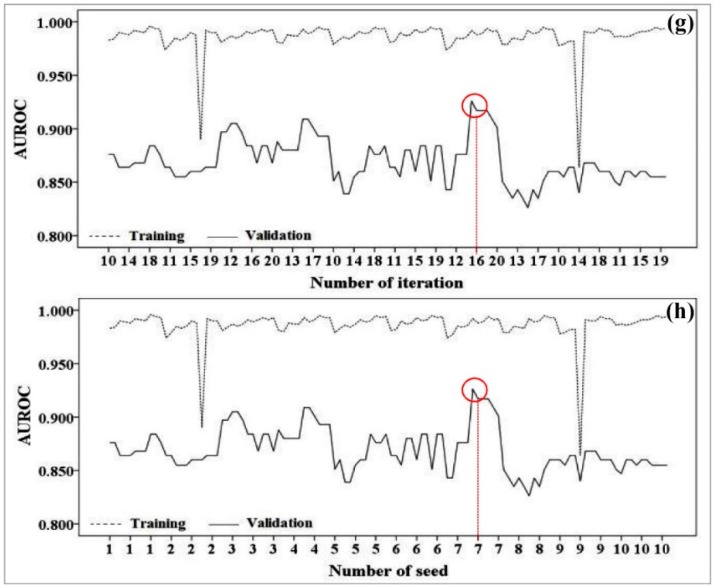
The trend of changes of the number of seed and iteration in the landslide modeling process: (**a**) optimum number of iteration for the combination of 60%/40% with the raster resolution of 10 m, (**b**) optimum number of seed for the combination of 60%/40% with the raster resolution of 10 m; (**c**) optimum number of iteration for the combination of 70%/30% with the raster resolution of 10 m, (**d**) optimum number of seed for the combination of 70%/30% with the raster resolution of 10 m; (**e**) optimum number of iteration for the combination of 80%/20% with the raster resolution of 20 m, (**f**) optimum number of seed for the combination of 80%/20% with the raster resolution of 20 m; (**g**) optimum number of iteration for the combination of 90%/10% with the raster resolution of 20 m, (**h**) optimum number of seed for the combination of 90%/10% with the raster resolution of 20 m.

According to Table 5 and Figure 5a–d, in the combination of 60%/40% with the raster resolution of 10 m, the best values for the number of iteration and seed were 16 and 7, respectively (Figure 5a,b), while, in the combination of 70%/30% with the raster resolution of 10 m, these values were 11 and 1 (Figure 5c,d). In addition, results showed that the values of 11 and 5 were the optimum values for the number of iteration and seed in the combination of 80%/20% with the raster resolution of 20 m (Figure 5e,f). In the combination of 90%/10% with the raster resolution of 20 m, these values were 15 and 7 (Figure 5g,h).

Based on the best selected values for the number of seed and iteration, modeling process using four ensemble models was performed, as shown in Table 6, Table 7, Table 8 and Table 9. Results of the combination of 60%/40% with the resolution of 10 m showed that in the training phase, the RS model had the highest prediction power based on sensitivity (0.938), specificity (0.900), accuracy (0.918) and ROC (0.974). The lowest prediction power belonged to the ADTree based on sensitivity and ROC, however, in terms of specificity, it belonged to RF. Accuracy index demonstrated that the prediction power of BA, RS, and ADTree were similar since all showed the lowest prediction power. The RS model obtained the highest prediction power for the validation phase based on specificity and accuracy, however, in terms of the sensitivity, the BA model outperformed other models. Overall, the RS model had the highest performance in comparison to the other models in the combination of 60%/40% with the resolution of 10 m (Table 6).

In the combination of 70%/30% with the raster resolution of 10 m, results concluded that the RS model outperformed the MB (0.964), BA (0.962), ADTree (0.951) and RF (0.948) models in the training phase, evaluated by sensitivity (0.974). In terms of the specificity, BA (1.000) and ADTree (1.000) showed the highest prediction power, followed by MB (0.987), RS (0.940) and RF (0.906). In terms of the accuracy, the BA (0.981) model had the higher performance, followed by the MB and ADTree (0.975), RS (0.957) and RF (0.926) models. Based on ROC, RS (0.997) outperformed the other models. MB in terms of sensitivity, BA and RF in terms of specificity, MB and ADTree in terms of accuracy, and RS in terms of ROC displayed the highest prediction capabilities for validation dataset. Overall, the results indicate that the RS model outperformed the other models in the combination of 70%/30% with the raster resolution of 10 m in the study area (Table 7).

The results in Table 8 show the performance of the ensemble models in the combination of 80%/20% with the raster resolution of 20 m. This table shows that the MB, BA, RF and ADTree models based on sensitivity (0.920), specificity (0.911) and accuracy (0.916) had the similar performance and outperformed the RS model in the training phase. However, in terms of the ROC, the MB model demonstrated the highest performance (0.988), followed by the RF (0.987), BA (0.974), RS (0.972) and ADTree (0.967) models. According to the validation dataset, the MB model outperformed the other models in terms of specificity (0.833), accuracy (0.864) and ROC (0.934), while, in terms of sensitivity, the ADTree model illustrated the highest prediction power (Table 8).

In Table 9, the results of the combination of 90%/10% with the raster resolution of 20 m reveal that the MB model had the highest performance in terms of sensitivity (0.948) and ROC (0.992) however the RF model outperformed the other models in terms of specificity (0.959) and accuracy (0.950) in the training phase. In the validation phase, although the MB, BA, RF and ADTree models showed the same performance and outperformed RS in terms of the sensitivity (0.909), specificity (0.909) and accuracy (0.909), MB displayed the highest prediction power in terms of the ROC (0.926), followed by the BA, RF, ADTree and RS models (Table 9).

### 5.4. Landslide Susceptibility Mapping

**Figure 6:**
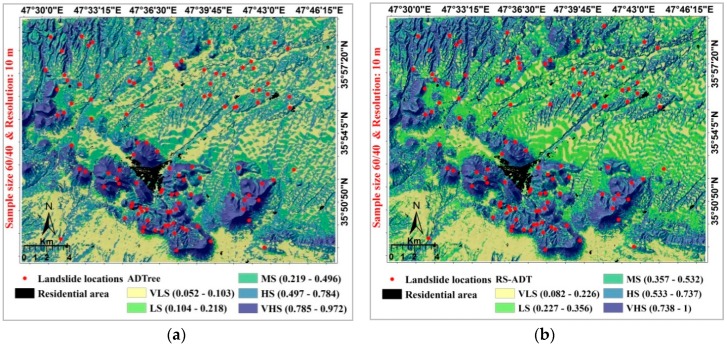

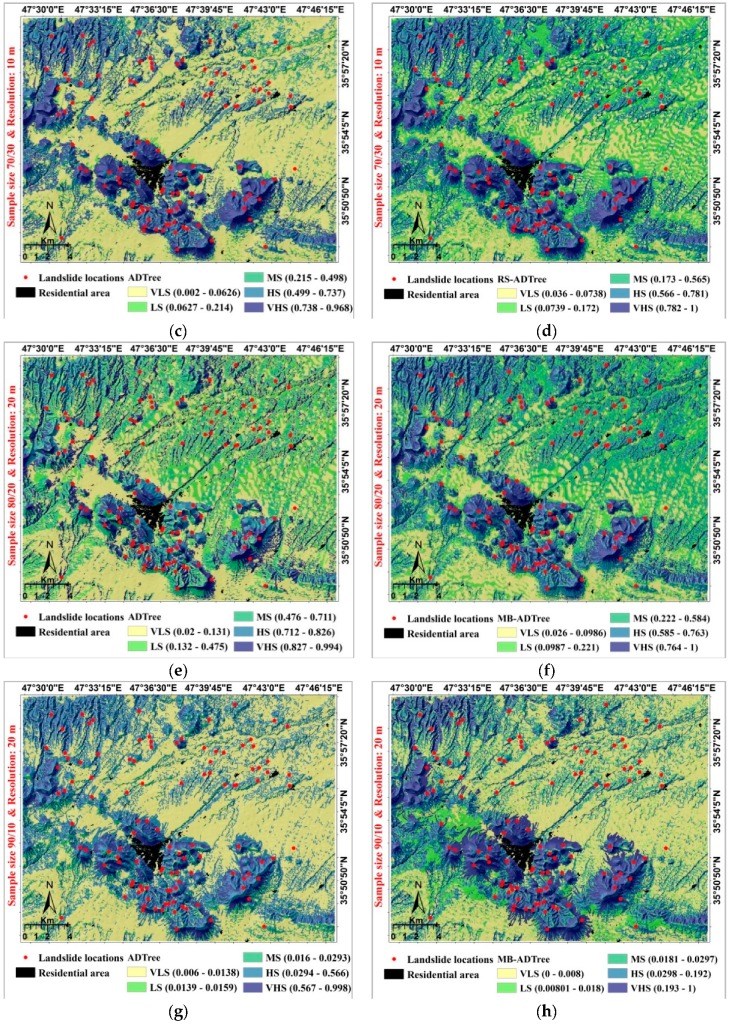
Landslide susceptibility mapping prepared by the ADTree model and its ensemble: (**a**) ADTree, sample size 60/40 & Resolution: 10 m; (**b**) RS-ADT, sample size 60/40 & Resolution: 10 m; (**c**) ADTree, sample size 70/30 & Resolution: 10 m; (**d**) RS-ADTree, sample size 70/30 & Resolution: 10 m; (**e**) ADTree, sample size 80/20 & Resolution: 20 m; (**f**) MB-ADTree, sample size 80/20 & Resolution: 20 m; (**g**) ADTree, sample size 90/10 & Resolution: 20 m; (**h**) MB-ADTree, sample size 90/10 & Resolution: 20 m.

After determining the best ensemble models, they were performed for generation of different landslide susceptibility maps. In the modeling process, for sample sizes of 60%/40% and 70%/30% with the raster resolution of 10 m, the RS model was selected as the most proper model for spatial prediction of landslides in the study area while the MB model was also selected as an acceptable model. At first step, models have been learned using the training dataset. The entire study area was then converted to a raster format and a unique value was assigned to each pixel based on the learned pattern, which called landslide probability index (LPI). These continuous indexes were classified based on the natural break classification scheme for developing the maps using different landslide probability occurrence or susceptibility. These classes of susceptibilities were very low (VLS), low (LS), moderate, high and very high (VHS), as shown in Figure 6.

### 5.5. Evaluation of Landslide Susceptibility Maps

**Figure 7:**
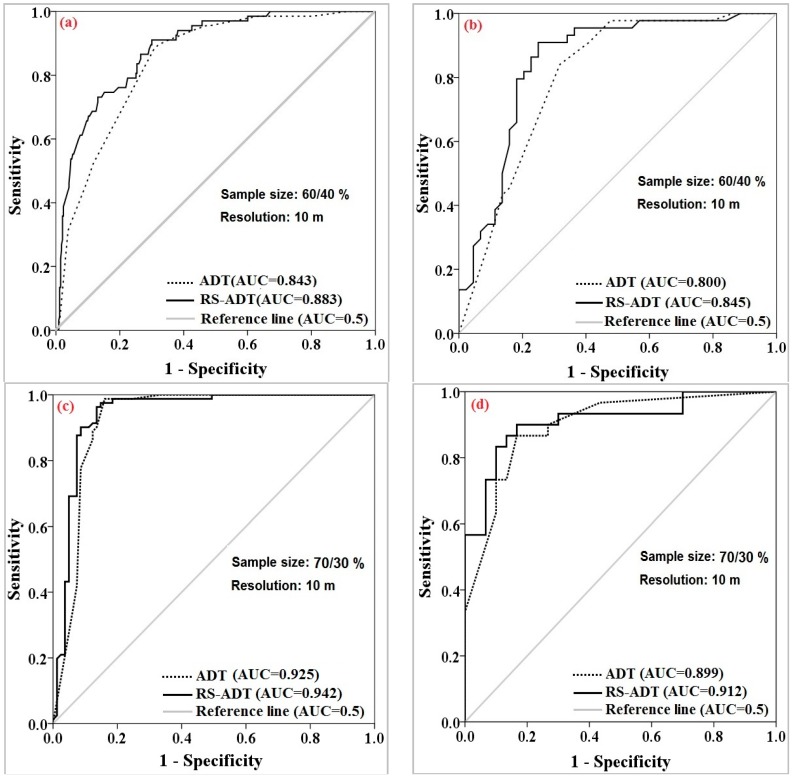

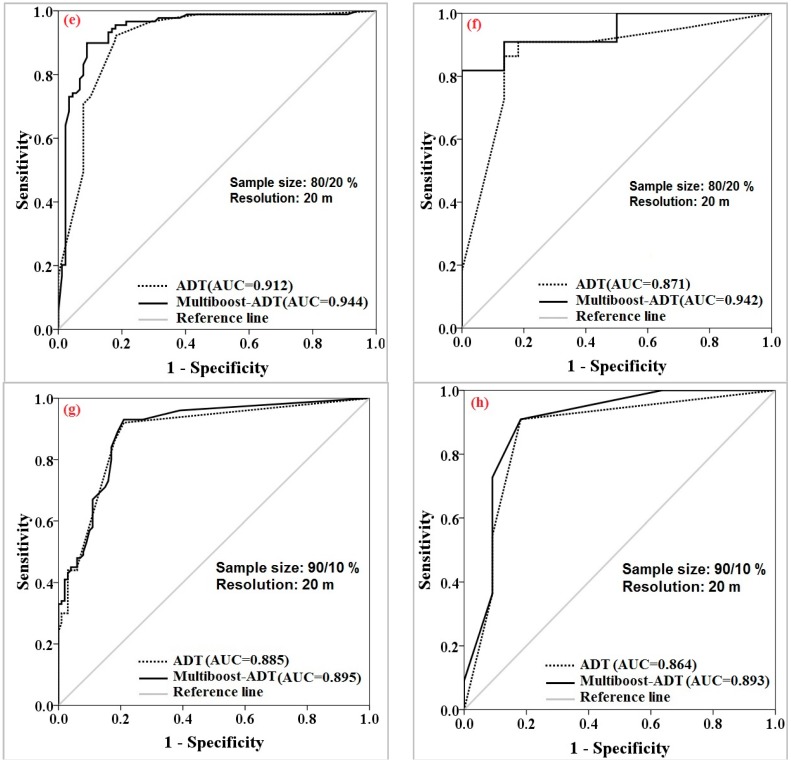
Model comparison and evaluation of the ADTree and its ensembles in different sample sizes and raster resolutions using: training dataset (**a**,**c**,**e**,**g**); and validation dataset (**b**,**d**,**f**,**h**).

Model validation was carried out using the ROC and AUC for both training and validation datasets (Figure 7a–h). In the combination of 60%/40% with the raster resolution of 10 m, the area under the ROC curve (AUROC) using training dataset (goodness-of-fit) by the ADTree as the base classifier and its ensemble of RS were 0.843 and 0.883, respectively (Figure 7a). Additionally, the AUROC using validation dataset (prediction accuracy) for the ADTree model was 0.800 and for RS was 0.845 (Figure 7b). In the combination of 70%/30% with the raster resolution of 10 m, the AUROC in ADTree and its ensemble of RS were 0.925 and 0.942, respectively (Figure 7c). These values for prediction accuracy were 0.899 and 0.912 (Figure 7d). Results shown in Figure 7e show that the AUROC using training dataset for ADTree was 0.912 while for its ensemble of MB was 0.944. Moreover, these values for prediction accuracy were 0.871 and 0.942 (Figure 7f). Ultimately, in the combination of 60%/40% with the raster resolution of 10 m, the AUC for ADTree and its ensemble of MB were 0.885 and 0.893, respectively (Figure 7g), while, for the validation dataset, these values were 0.864 and 0.893 (Figure 7h).

Overall, results of comparison and validation of ADTree and its ensembles for different sample sizes and raster resolutions indicated that the MB model had the highest prediction power for the combination of 80%/20% with the resolution of 20 m, followed by the combination of 70%/30% with the resolution of 10 m using the RS model, the combination of 90%/10% with the resolution of 10 m by the MB model and the combination of 60%/40% with the resolution of 10 m by the RS model.

## 6. Discussion

Since many methods and modeling techniques have been developed for preparing landslide susceptibility assessment, increasing the performance of landslide models has been more attempted by landslides researchers [72]. In other words, the goodness-of-fit and prediction accuracy of new machine learning algorithms have been questioned in landslide modeling [17]. Hence, the main objective of this study was to check the performance of the ADTree algorithm as a decision tree algorithm in combination with four Meta classifiers/machine learning ensembles: MB, Bagging, RF and Random subspace RS for landslide susceptibility mapping. What is more predominated in this study is the design of two scenarios: (i) the combination of two sample sizes including 60%/40% and 70%/30% with a resolution of 10 m; and (ii) the combination of two sample sizes including 80%/20% and 90%/10% with a resolution of 20 m for training and validation datasets. It is safe to say that, according to the literature review, ADTree and its ensembles have rarely been used at Bijar City in Kurdistan Province of Iran.

The results of this study proved that Meta classifiers were improved the goodness-of-fit and prediction accuracy of ADTree as a single-based algorithm (base classifier) in the two scenarios. Our findings were reasonable because the ensemble classifiers decreased the bias, variance, and over-fitting problems in landslide modeling to enhance the performance of base classifier [19]. These results are in agreement with those in [19,22,73,74], which report that ensemble models lead to increasing the performance of the singles-based models. The findings also include that, in the first scenario, the RS ensemble model had the highest goodness-of-fit (AUC = 0.942) and prediction accuracy (AUC = 0.912). Shirzadi et al. [22] expressed that the RS ensemble model can well detect the weakness of the NBTree base classifier in determining landslide locations around Bijar City, Kurdistan Province, Iran. In the second scenario, results indicate the superiority of the MB ensemble model in both goodness-of-fit (AUC = 0.944) and prediction accuracy (AUC = 0.942). Among all four machine learning ensemble models, ADTree with the Multiboost model (MB-ADTree) acquired the highest improvement. It is because the MB ensemble model has more ability for reducing the bias, variance, and over-fitting problems compared to other ensemble methods. This finding was exactly similar to that found by Pham et al. [74] who declared that MB is a powerful ensemble technique in comparison to Adaboost, Bagging, Dagging, Rotation Forest and Random Subspace models for spatial predation of landslides. It is remarkable that the success of a landslide model depends completely on the training dataset with lower noise and over-fitting problems. In other words, selecting a proper training dataset including landslide and non-landslide locations in conjunction with all conditioning factors is a critical issue in landslide modeling. Landslides and non-landslide locations were randomly selected and the most important conditioning factors to assess their predictive capability for modeling were extracted using the information gain ratio (IGR) technique for the two scenarios.

This technique led to the selection of the best factors with low noise for modeling process [75,76]. Results show that, in the combination of 60%/40% with the resolutions of 10 m and 20 m, among twenty factors, only five factors including slope angle, TWI, aspect, LS, and profile curvature were more effective. Our findings also indicate that, in the combinations of 70%/30%, 80%/20% and 90%/10% with the resolutions of 10 and 20 m, twelve factors were important for landslide modeling including slope angle, TWI, aspect, LS and profile curvature, plan curvature, elevation, curvature, Land use, rainfall, SPI, and solar radiation. Ineffective factors were removed from the modeling process due to having average merit equal to 0. Moreover, slope angle and TWI were the two most significant factors in the study area contributing in landslide occurrence. Therefore, our findings reveal that, with removing the factors with low predictive capability, the performance of the models increased. It was found that, to achieve a powerful and capable ensemble model, the parameters affecting the results of modeling should be correctly determined such as number of seed and number of iteration. Therefore, it is necessary to optimize these parameters to obtain the best performance of theses ensemble models.

## 7. Conclusions

The core of this study was to present a hybrid approach of ADTree and different ensemble algorithms (Multiboost, Bagging, Rotation forest and Random subspace) to construct different ensemble models including ADTree-MB, ADTree-Bagging, ADTree-RF, and ADTree-RS for the development of landslide susceptibility maps in Bijar City, Kurdistan province, Iran. Performance of these models was evaluated using sensitivity, specificity, accuracy, Kappa and RMSE measures. We found that the resolution of 10 m obtained more performance for sample sizes of 60%/40% and 70%/30% while the best performance was acquired by the resolution of 20 m and sample sizes of 80%/20% and 90%/10%. Additionally, we found that, for sample sizes of 60%/40% and 70%/30%, The RS-ADTree outperformed other ensemble models, while the MB-ADTree had the most prediction accuracy in comparison to other ensemble models for sample sizes of 80%/20% and 90%/10%. It implies that the RS and MB models could more decrease the noise and over-fitting problems and hence they produced better results than the other ensemble models. Moreover, among all sample sizes and raster resolutions, the MB-ADTree models (a raster resolution of 20 m and a sample size of 80%/20%) outperformed and outclassed other ensemble models. Therefore, MB-ADTree model could be efficiently used for predicting landslide susceptibility. This model could also serve environmental managers in decision-making and developing pro-active environmental management policies in landslide-prone regions.

## Figures and Tables

**Table 1:** Landside conditioning factors and their classes for landslide modeling in Bijar City.

	No.	Landslide Causal Factors	Classes
Topographic factors	1	Slope (^o^)	(1) 0–5; (2) 5–10; (3) 10–15; (4) 15–20; (5) 20–25; (6) 25–30; (7) 30–45; (8) >45
	2	Aspect	(1) Flat; (2) North; (3) Northeast; (4) East; (5) Southeast; (6) South; (7) Southwest; (8) West; (9) Northwest
	3	Elevation (m)	(1) 1573–1700; (2) 1700–1800; (3) 1800–1900; (4) 1900–2000; (5) 2000–2100; (6) 2100–2200; (7) 2200–2300; (8) 2300–2400; (9) >2400
	4	Curvature (m^−1^)	(1) [(−12.5)–(−1.4)]; (2) [(−1.4)–(−0.4)]; (3) [(−0.4)–(−0.2)]; (4) [(−0.2)–0.9]; (5) [0.9–2.5]; (6) [2.5–15.6]
	5	Plan curvature (m^−1^)	(1) [(−6.7)–(−0.8)]; (2) [(−0.8)–(−0.2)]; (3) [(−0.2)–0]; (4) [0–0.4]; (5) [0.4–1.1]; (6) [1.1–10.4]
	6	Profile curvature (m^−1^)	(1) [(−10.7)–(−1.7)]; (2) [(−1.7)–(−0.7)]; (3) [(−0.7)–(−0.2)]; (4) [(−0.2)–0.2]; (5) [0.2–0.9]; (6) [0.9–7.5]
	7	STI	(1) 0–7; (2) 7–14; (3) 14–21; (4) 21–28; (5) 28–35; (6) 35–42
Hydrological factors	8	Rainfall (mm)	(1) 263–270; (2) 270–300; (3) 300–330; (4) 330–360; (5) 360–390; (6) 390–420; (7) 420–450
	9	Annual solar radiation (h)	(1) 3.015–6.563; (2) 5.563–6.747; (3) 6.747–6.849; (4) 6.849–6.930; (5) 6.930–7.073; (6) 7.073–7.236; (7) 7.236–8.215
	10	SPI	(1) 0–998; (2) 998–6986; (3) 6986–19,961; (4) 19,961–45,911; (5) 45,911–101,803; (6) 101,803–255,505
	11	TWI	(1) 1–3; (2) 3–4; (3) 4–6; (4) 6–8; (5) 8–9; (6) 9–11
	12	Distance to Rivers (m)	(1) 0–50; (2) 50–100; (3) 100–150; (4) 150–200; (5) >200
	13	River density (km/km^2^)	(1) 0–1.9; (2) 1.9–3.2; (3) 3.2–4.2; (4) 4.2–5.2; (5) 5.2–6.3; (6) 6.3–7.8; (7) 7.8–13.2
Lithological factors	14	Lithology	(1) Quaternary (2) Tertiary (3) Cretaceous
	15	Distance to Faults (m)	(1) 0–200; (2) 200–400; (3) 400–600; (4) 600–800; (5) 800–1000; (6) >1000
	16	Fault density (km/km^2^)	(1) 0–0.3; (2) 0.3–0.8; (3) 0.8–1.2; (4) 1.2–1.7; (5) 1.7–2.1; (6) 2.1–2.5; (7) 2.5–3.2
Land Cover Factors	17	Land use	(1) Residential area (2) Arable land (dry faring and cultivated lands); (3) Wood land; (4) Grassland; (5) Barren land
	18	NDVI	(1) [(−0.23)–(−0.061)]; (2) [(−0.061)–(−0.0081)]; (3) [(−0.0081)–(0.060)]; (4) [(0.060)–0.14]; (5) [0.14–0.24]; (6) [0.24–0.41]; (7) [0.41–0.73]
Anthropogenic factors	19	Distance to Roads (m)	(1) 0–50; (2) 50–100; (3) 100–150; (4) 150–200; (5) >200
	20	Road density (km/km^2^)	(1) 0–0.0013; (2) 0.0013–0.0027; (3) 0.0027–0.0041; (4) 0.0041–0.0055; (5) 0.0055–0.0069; (6) 0.0069–0.0083; (7) 0.0083–0.0097

**Table 2:** Factor selection based on the information gain ration techniques.

Conditioning Factors	10 m								20 m							
	60%/40%		70%/30%		80%/20%		90%/10%		60%/40%		70%/30%		80%/20%		90%/10%	
	AM	R	AM	R	AM	R	AM	R	AM	R	AM	R	AM	R	AM	R
Slope angle	0.105	2	0.482	1	0.509	1	0.484	1	0.135	2	0.655	1	0.459	1	0.481	1
TWI	0.142	1	0.597	2	0.427	2	0.409	2	0.142	1	0.482	2	0.428	2	0.409	2
Aspect	0.071	3	0.065	10	0.058	11	0.088	6	0.071	3	0.072	9	0.065	7	0.085	6
STI	0.064	4	0.195	4	0.172	4	0.186	4	0.064	4	0.195	4	0.173	3	0.186	3
Profile curvature	0.005	5	0.042	12	0.094	7	0.031	12	0	-	0.032	12	0.011	12	0	-
Plan curvature	0	-	0.221	3	0.174	3	0.191	3	0	-	0.440	3	0.172	4	0.167	4
Elevation	0	-	0.096	7	0.086	8	0.095	5	0	-	0.096	7	0.059	9	0.095	5
Curvature	0	-	0.114	5	0.106	5	0.085	7	0	-	0.065	10	0.022	11	0.046	11
Land use	0	-	0.064	9	0.058	11	0.050	11	0	-	0.080	8	0.058	10	0.070	8
Rainfall	0	-	0.051	11	0.064	10	0.057	10	0	-	0.051	11	0.065	8	0.057	10
SPI	0	-	0.070	8	0.075	9	0.071	9	0	-	0.116	6	0.076	6	0.071	9
Solar radiation	0	-	0.099	6	0.092	6	0.081	8	0	-	0.119	5	0.077	5	0.076	7

AM, Average Merit; R, Rank.

**Table 3:** Model performance using training dataset and ADTree algorithm.

Raster Resolution (m)	10				20			
Sample Size (%)	60%/40%	70%/30%	80%/20%	90%/10%	60%/40%	70%/30%	80%/20%	90%/10%
Statistic Measures								
TP	60	77	85	91	55	72	81	89
TN	47	81	76	89	48	78	82	92
FP	7	0	4	9	12	9	8	11
FN	20	4	13	11	19	3	7	8
SST %	0.750	0.951	0.867	0.892	0.743	0.960	0.920	0.918
SPF %	0.870	1.000	0.950	0.908	0.800	0.897	0.911	0.893
ACC %	0.799	0.975	0.904	0.900	0.769	0.926	0.916	0.905
Kappa	0.597	0.950	0.809	0.800	0.537	0.851	0.831	0.810
RMSE	0.351	0.157	0.291	0.300	0.407	0.239	0.273	0.298

**Table 4:** Model performance using validation dataset and ADTree algorithm.

Raster Resolution (m)	10				20			
Sample Size (%)	60%/40%	70%/30%	80%/20%	90%/10%	60%/40%	70%/30%	80%/20%	90%/10%
Statistic Measures								
TP	26	27	17	10	19	27	18	10
TN	37	23	19	10	25	22	20	10
FP	18	3	5	1	35	3	4	1
FN	7	7	3	1	9	8	1	1
SST %	0.788	0.794	0.850	0.909	0.679	0.771	0.947	0.909
SPF %	0.673	0.885	0.792	0.909	0.417	0.880	0.833	0.909
ACC %	0.716	0.833	0.818	0.909	0.500	0.817	0.884	0.909
Kappa	0.631	0.666	0.636	0.818	0.572	0.633	0.727	0.818
RMSE	0.363	0.182	0.390	0.331	0.484	0.256	0.342	0.309

**Table 5:** The optimal values of the number of iteration and seed for different sample sizes and raster resolutions using ensemble models.

Ensemble Models	90%/10% and Resolution 20 m		80%/20% and Resolution 20 m		70%/30% and Resolution 10 m		60/410% and Resolution 10 m	
	S	I	S	I	S	I	S	I
MB	7	15	5	11	3	10	1	14
BA	3	10	4	10	6	10	8	10
RS	4	10	8	10	1	11	7	16
RF	6	15	3	13	5	13	1	14

I, iteration; S, seed.

**Table 6:** Results of ensembles modeling by combination of 60%/40% and raster resolution of 10 m.

Criteria	ADTree		RF		RS		BA		MB	
	T	V	T	V	T	V	T	V	T	V
True positive	60	26	46	26	60	30	48	27	52	29
True negative	47	37	61	36	63	36	59	37	63	33
False positive	7	18	21	18	7	14	19	17	15	15
False negative	20	7	6	8	4	8	8	7	4	11
Sensitivity	0.750	0.788	0.885	0.765	0.938	0.789	0.857	0.794	0.929	0.725
Specificity	0.870	0.673	0.744	0.667	0.900	0.720	0.756	0.685	0.808	0.688
Accuracy	0.799	0.716	0.799	0.705	0.918	0.750	0.799	0.727	0.858	0.705
AUROC	0.864	0.737	0.907	0.796	0.974	0.791	0.889	0.788	0.940	0.756

T, training dataset; V, validation dataset.

**Table 7:** Results of ensembles modeling by combination of 60%/40% and raster resolution of 10 m.

Criteria	ADTree		RF		RS		BA		MB	
	T	V	T	V	T	V	T	V	T	V
True positive	77	27	73	28	76	28	75	28	80	28
True negative	81	23	77	22	79	21	78	21	78	22
False positive	0	3	8	2	5	2	6	2	1	8
False negative	4	7	4	8	2	9	3	9	3	2
Sensitivity	0.951	0.794	0.948	0.778	0.974	0.757	0.962	0.757	0.964	0.933
Specificity	1.000	0.885	0.906	0.917	0.940	0.913	1.000	0.913	0.987	0.733
Accuracy	0.975	0.833	0.926	0.833	0.957	0.817	0.981	0.817	0.975	0.833
AUROC	0.979	0.862	0.984	0.898	0.997	0.901	0.983	0.893	0.996	0.892

T, training dataset; V, validation dataset.

**Table 8:** Results of ensembles modeling by combination of 80%/20% and raster resolution of 20 m.

Criteria	ADTree		RF		RS		BA		MB	
	T	V	T	V	T	V	T	V	T	V
True positive	81	18	81	19	78	18	81	18	81	18
True negative	82	20	82	20	82	20	82	20	82	20
False positive	8	4	8	3	11	4	8	4	8	4
False negative	7	1	7	2	7	2	7	2	7	2
Sensitivity	0.920	0.947	0.920	0.905	0.918	0.900	0.920	0.900	0.920	0.900
Specificity	0.911	0.833	0.911	0.870	0.882	0.833	0.911	0.833	0.911	0.833
Accuracy	0.916	0.884	0.916	0.886	0.899	0.864	0.916	0.864	0.916	0.864
AUROC	0.967	0.903	0.987	0.937	0.972	0.926	0.974	0.926	0.988	0.934

T, training dataset; V, validation dataset.

**Table 9:** Results of ensembles landslide modeling using combination of 90%/10% and raster resolution of 20 m.

Criteria	ADTree		RF		RS		BA		MB	
	T	V	T	V	T	V	T	V	T	V
True positive	89	10	96	10	88	10	87	10	92	10
True negative	92	10	94	10	92	9	93	10	95	10
False positive	11	1	4	1	12	1	13	1	8	1
False negative	8	1	6	1	8	2	7	1	5	1
Sensitivity	0.918	0.909	0.941	0.909	0.917	0.833	0.926	0.909	0.948	0.909
Specificity	0.893	0.909	0.959	0.909	0.885	0.900	0.877	0.909	0.922	0.909
Accuracy	0.905	0.909	0.950	0.909	0.900	0.864	0.900	0.909	0.935	0.909
AUROC	0.957	0.876	0.983	0.913	0.968	0.884	0.968	0.921	0.992	0.926

T, training dataset; V, validation dataset.
